# Influence of UV Radiation on the Appearance Quality of Fair-Faced Concrete and Mitigation Approaches

**DOI:** 10.3390/ma18092039

**Published:** 2025-04-29

**Authors:** Ao Wu, Jia Ke, Zhijie Liu, Zhonghe Shui

**Affiliations:** 1 International School of Materials Science and Engineering, Wuhan University of Technology, Wuhan 430070, China; 289451@whut.edu.cn (A.W.); 258732@whut.edu.cn (J.K.);; 2 State Key Laboratory of Silicate Materials for Architectures, Wuhan University of Technology, Wuhan 430070, China

**Keywords:** fair-faced concrete, appearance quality, UV radiation, anti-UV admixture, pore distribution

## Abstract

Fair-faced concrete has garnered substantial attention in recent years owing to its aesthetic appeal and eco-friendly attributes. However, as a construction material, its long-term performance is highly dependent on its service environment, particularly ultraviolet (UV) radiation. This research focuses on examining the influence of UV exposure and managing the admixtures employed in concrete and investigating the effects of UV radiation on the appearance quality, pore distribution, and micro-composition of fair-faced concrete. Results indicate that UV radiation enhances moisture evaporation, increases surface and bulk porosity, and accelerates carbonation and early hydration reactions, forming more calcite on the surface. These factors degrade the appearance quality of fair-faced concrete. To mitigate UV-aging damage, two common anti-UV admixtures, nano-silica (NS) and water-based fluorocarbon paint (FC), were evaluated. Results show that both admixtures effectively improve the UV-resistance of fair-faced concrete, particularly when combined. The FC+NS group reduced the surface glossiness loss rate from 28.63% to 12.95% after 28 days of UV exposure, with surface porosity and maximum pore diameter recorded at 0.157% and 3.66 mm, respectively, indicating excellent appearance quality. These findings underscore the potential of these admixtures, both individually and in combination, to enhance the UV resistance of fair-faced concrete, sustaining its durability under prolonged UV exposure.

## 1. Introduction

Fair-faced concrete (FFC) is a building material combining aesthetic appeal, functionality, and environmental benefits [1,2]. It has a smooth and even surface, as well as uniform color, which eliminates the need for secondary decoration or painting. This directly showcases the texture and grain of the concrete itself [3]. Compared to traditional construction materials, FFC remarkably lowers resource consumption and generates less construction waste [4,5,6,7]. It is widely used in modern architecture, particularly favored by many international architects for its simple and elegant style. Therefore, the appearance quality of FFC is a key factor in evaluating its overall quality and how to improve and maintain its appearance quality is particularly important [8].

Recent years have seen research on the surface quality of FFC primarily focus on the improvement effects of various mineral admixtures on surface quality, as well as the development of testing methods and standard systems for surface quality [9,10]. For instance, Luan et al. [11] discovered that fly ash particles can act as a lubricant during concrete mixing, enhancing the mix proportion and allowing better filling of formwork and dispersion of air bubbles, which lowers color differences and air bubbles in FFC to some extent [12,13,14,15]. However, other studies have shown that excessive fly ash can make the local microstructure more dense, resulting in uneven dark spots and significant surface color differences, degrading FFC surface quality [16,17]. When evenly distributed and combined with the cementitious material, the low-density and extensive specific surface area of limestone powder can enhance the interfacial properties between the cementitious material and water, thereby improving water retention in concrete, according to Wang et al. [18,19]. This enhancement results in the improved appearance quality of FFC. The majority of the limestone powder is composed of smooth calcite, which, as demonstrated by microscopic research, creates optimal conditions for the nucleation and growth of C-S-H gel during hydration [20,21,22,23].

With the development of digital processing technology, testing methods for the appearance performance of FFC have gradually shifted from visual subjective judgment to digital processing [2,24,25,26]. For instance, Shi et al. [27] summarized the process of evaluating the appearance quality of FFC through four steps: image acquisition, grayscale processing, bubble analysis, and data evaluation. They noted that in image processing technology, the image acquisition stage is most susceptible to external factors and shooting conditions.

In reality, as a building material, FFC is vulnerable to various natural environmental influences, such as long-term sunlight exposure, acid rain corrosion, and weathering [28]. These factors result in increased surface abrasions and a significant decline in surface gloss, making it aesthetically unappealing [29,30]. More critically, prolonged erosion from natural environments can cause the structural deterioration of the concrete, including cracking, increased porosity and pore size distribution, and a significant reduction in strength and durability [31,32,33]. These factors collectively impact the actual lifespan of FFC [34]. However, systematic investigations into the long-term performance of FFC under natural weathering conditions remain scarce within the field of construction materials research.

Sunlight is the most common working environment for FFC, inevitably exposing it to prolonged UV radiation [35,36,37]. UV rays, a form of high-energy electromagnetic waves, have the potential to break chemical bonds in many materials, triggering a series of reactions, like free radical reactions and oxidation reactions. These reactions can lead to material degradation and a decline in performance [38,39,40]. Most current research focuses on the degradation of materials like asphalt, epoxy resins, and fiber-reinforced polymers used in concrete under UV radiation [41,42,43,44]. However, recent studies have made new progress in understanding the changes in cement-based materials under UV light [45,46,47,48]. For instance, Li et al. [49] found that UV radiation accelerates the carbonation reaction in cement-based materials, facilitating the formation of stable calcium carbonate crystals like calcite. This stable crystal formation not only increases the density and strength of the material but also improves its macro and micro mechanical properties. Wang et al. [50] discovered that under strong UV radiation, the porosity and mesoporosity of cement-based materials significantly increase while the degree of hydration decreases and drying shrinkage deformation increases. UV radiation primarily influences both the physical and chemical properties of the surface layer structure of concrete, resulting in alterations to the composition and morphology of this layer. The surface quality is critical for fair-faced concrete, as it significantly affects the longevity of its use. However, the impact of UV radiation on the appearance quality of fair-faced concrete has not been extensively acknowledged. Furthermore, strategies to effectively mitigate the effects of UV radiation on fair-faced concrete have been infrequently addressed. Consequently, investigating UV radiation effects on both the surface characteristics and chemical composition of fair-faced concrete is critical for enhancing its long-term durability and maintaining aesthetic performance under natural weathering conditions.

In summary, it is essential to study the effects of UV light exposure on the appearance properties of FFC. With the increasing demand for high-quality surfaces as well as durability in FFC in production and application, this research also examines the inhibitory effects of two common UV-resistant admixtures, nano-silica and fluorocarbon paint, on UV radiation, focusing on surface properties and chemical composition. Therefore, this experiment investigates the effects of UV light exposure on the appearance quality, mass loss, pore size distribution, and microscopic composition of mortar with different cement mineral admixtures. There were several objectives: (1) to understand the impact of UV radiation on the appearance quality of mortar with different admixtures; (2) to research the effects of UV exposure on the hydration process, pore distribution, and carbonation extent of fair-faced concrete mortar; (3) to investigate the association between the surface quality and microstructural properties of fair-faced concrete under UV radiation; (4) to confirm that two different admixtures are capable of mitigating the UV aging on fair-faced concrete.

## 2. Materials and Methods

### 2.1. Materials

The cement used was PW52.5 produced by Aalborg White Cement Company (Aalborg, Denmark) with a specific surface area of 1.18 m^2^/g. Metakaolin (MK) supplied by Yangzhou Dilan Chemical Raw Materials Co., Ltd. (Yangzhou, China) had a specific surface area of 1.86 m^2^/g. Limestone powder (LP) produced by a lime plant had a specific surface area of 3.14 m^2^/g. Two admixtures, nano-silica (20 ± 5 nm) and water-based fluorocarbon paint, were bought from Shanghai Macklin Biochemical Technology Co., Ltd. (Shanghai, China) and Shanghai Jinsidi Industrial Co., Ltd. (Shanghai, China), respectively, with the nano-silica having a specific surface area of 450 m^2^/g. Fresh tap water was employed as the water source. Both quartz sand and quartz powder were sourced from Minghai Quartz Sand Factory (Zhengzhou, China), with quartz sand being 30 mesh and quartz powder available in four types: 50 mesh, 100 mesh, 150 mesh, and 350 mesh, denoted as A, B, C, and D, respectively. The water reducer was the 540 P polycarboxylate superplasticizer from Sika with a water reduction rate of approximately 22%. The defoamer used was the FKXP-1 defoamer from Shanxi Feike New Materials Technology Co., Ltd. (Taiyuan, China). The chemical composition of the PW, MK, and LP was analyzed using X-ray fluorescence (XRF), with the chemical composition of each raw material shown in Table 1.

### 2.2. Experimental Arrangements

#### 2.2.1. Mix Proportions

The specifications of JGJ169-2009 [1,25] (Technical Specification for Application of Fair-faced Concrete) were followed to calculate the particle gradation of raw materials for meeting high-density requirements. The MAA model was used to adjust the water-cement ratio [51,52,53]. The optimal water–cement ratio (0.195) was determined based on wet packing density [54,55] and the mix design for the control group is presented in Table 2. The FC group, NS group, and FC+NS group incorporated 1% fluorocarbon paint, 0.5% nano-silica, and a combination of 1% fluorocarbon paint and 0.5% nano-silica, respectively, without changing the original mix ratio of the negative control (NC) group. The dosage of the admixture was determined via preliminary experiments, which indicated that the FFC with this proportion exhibited the highest surface glossiness and the lowest surface porosity.

#### 2.2.2. Mixing Method

The raw materials were thoroughly mixed before being added to a JJ-5 mortar mixer (Nanjing T-Bota Scietech Instruments & Equipment Co., Ltd., Jiangsu, China) for thorough mixing. Water, water reducer, defoamer, and admixtures were then slowly incorporated, followed by 180 s of slow mixing and 60 s of fast mixing. The mixed mortar was quickly poured into a customized iron mold with dimensions of 200 × 100 × 15 mm, which had been lined with a 0.5 mm smooth PET film. The molds were placed on a vibrating table (Hebei Junfan Experimental Instruments Co., Ltd., Cangzhou, China) until no more air bubbles were visible on the mortar surface. After molding, all specimens were placed in a standard curing condition at 20 ± 2 °C with a relative humidity above 95%. Following 7 days of curing, all specimens were removed, and the sample surfaces were dried. The cured samples exhibit minimal surface pores and a smooth, glossy finish. The mortar was then tested for its appearance quality, mass loss, and microstructural properties in various experimental environments.

#### 2.2.3. UV Radiation System Configuration

The design of the UV radiation system was based on annual average radiation statistics from regions with high UV radiation, as cited in the literature, where the total radiation typically ranges from 6000 to 8000 MJ/m^2^ [56,57]. The aging chamber (Shaoxing Zhicheng Instrument Co., Ltd., Zhejiang, China) is equipped with four UVA340 lamps, with a wavelength range of 320–400 nm, suitable for simulating sunlight testing. As provided by the manufacturer of the UV irradiance meter, when the UV lamp is positioned 25 cm from the mortar specimen surface, the test irradiance *T* is 33 W/m^2^. The test irradiance *T* (W/m^2^) can be derived using the following formula:

(1) $$T=\frac{T^{\prime }}{L^{2}},$$

where *T′* represents the theoretical irradiance (W/m^2^) and *L* denotes the distance between the sample surface and the UV light source (cm). Based on this formula, when the distance between the UV light source and the surface of the specimen slice is set to 10 cm, the test irradiance *T* in the UV aging environment is calculated to be 206.3 W/m^2^. The radiation dose *Q* (MJ/m^2^) can then be calculated using the following formula:

(2) Q=T×t

where T represents the test irradiance (W/m^2^) and t represents the test duration (s) so the daily average irradiance of the UV simulation environment is calculated to be 17.8 MJ/m^2^. This value lies within the observed daily average UV irradiance range of 16.5–21.2 MJ/m^2^, which is typically found in regions with high UV radiation levels, such as those with irradiance values ranging from 6000 (16.5 MJ/m^2^) to 8000 (21.2 MJ/m^2^). The radiation intensity of the ultraviolet environment simulated in this experiment is higher than that of the majority of regions worldwide. Consequently, the configuration of the UV radiation experimental setting is considered valid.

#### 2.2.4. Experimental Conditions

**Figure 1:**
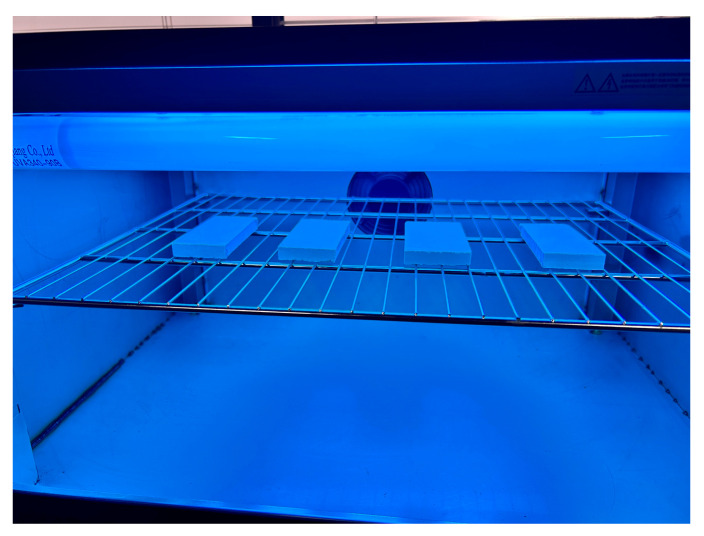
The UV radiation simulation environment.

Two experimental environments were established to analyze the influences of UV radiation on the appearance quality, mass loss, and microstructure of the samples. Additionally, the impacts of the addition of NS and FC on the appearance quality and microstructure were also analyzed. The particular parameters of the two environments are outlined below: (1) the standard environment had a relative humidity of 60 ± 5% as well as a temperature of 20 ± 2 °C; (2) the UV radiation environment had a relative humidity of 60 ± 5% as well as a temperature of 20 ± 2 °C. The UV radiation setup involved a UV lamp positioned 10 cm above the surface of the mortar specimens. Samples were arranged in parallel and equidistantly to ensure that the radiation intensity received on the surface was uniform. Figure 1 illustrates the setup of the UV radiation environment.

### 2.3. Test Methods

#### 2.3.1. Surface Glossiness

Gloss, as a surface characteristic of an object, depends on the surface’s ability to specularly reflect light. Glossiness, a physical quantity, is used to evaluate a material’s surface’s reflective ability under specified geometric conditions [58,59,60].

**Figure 2:**
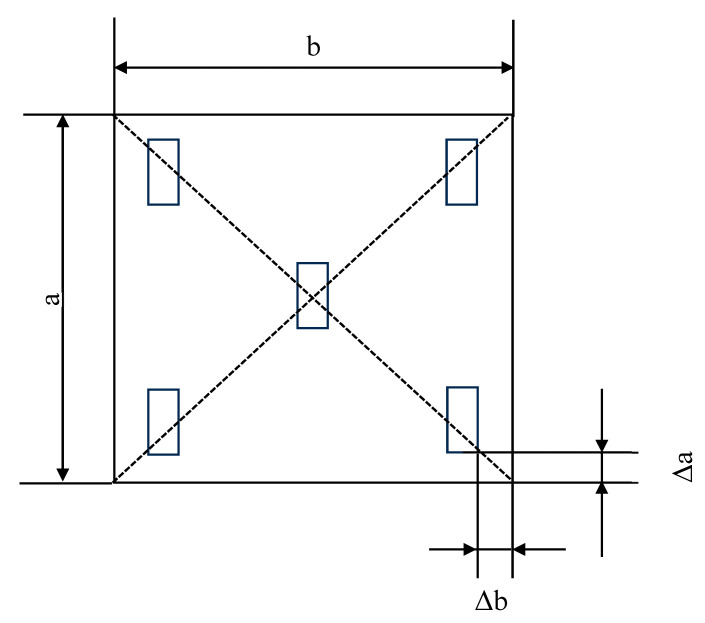
The diagram depicts a gloss meter test.( a and b represent the side lengths of the samples).

According to GB/T 13891-2008 [58] (test method of specular gloss for decorative building materials (ISO2813:1994 method)), the glossiness of each sample surface was measured using a gloss meter produced by 3NH Corporation (Shenzhen Sanenshi Technology Co., Ltd., Guangdong, China), model HG60. Measurements were taken at the center and four corners of the surface, as shown in Figure 2, and the average of the five test results was taken as the glossiness of the test block. Here, Δa and Δb represent the distances from the edges of the gloss meter to the edges of the test block, with a length of 10 mm. The calculation results were accurate to 0.1 gloss units.

#### 2.3.2. Surface Porosity

A Fuji X-M5 digital camera (Fujifilm Corporation, Tokyo, Japan) with 26.1-megapixel resolution was utilized to image the sample surface. The specimen surface dimensions were 50 mm × 100 mm and the small collection area dictated a 50 cm lens-to-specimen distance during image acquisition. Uniform, sufficient lighting from a single source was ensured for consistent image quality and a 0.1 mm precision ruler was placed within the collection area on the surface of samples with the lens positioned directly above and in front of it. Image Pro Plus software (Image Pro Plus 6.0) was employed to explore surface pore distribution on the fair-faced concrete surface.

**Figure 3:**
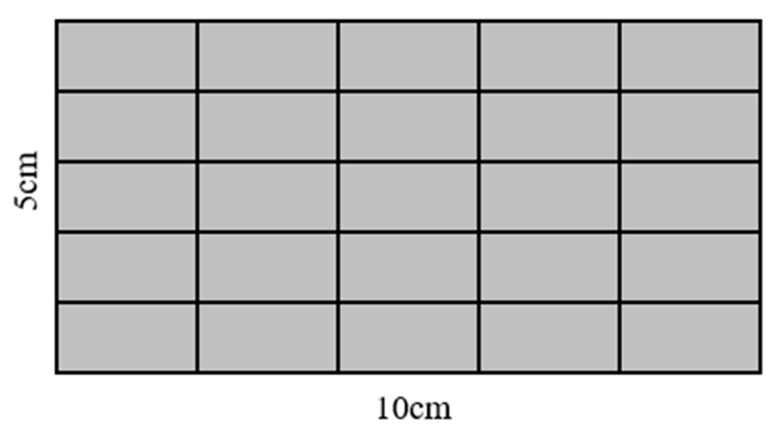
Surface division diagram of test blocks.

Maximum pore diameter and surface pore area ratio were directly exported from the Image Pro Plus software The standard deviation of the pore distribution was calculated by dividing the surface into 25 equal sections, as shown in Figure 3. Image Pro Plus was employed to calculate the surface pore area ratio for each of these 25 parts and the standard deviation was obtained from the standard deviation of these 25 surface pore area ratios. A higher calculated standard deviation indicates a more uneven pore distribution.

#### 2.3.3. Roughness

Surface roughness and topography of the samples were assessed using a laser scanning confocal microscope (LSCM) from Zeiss, Suita, Japan, model LSM 800/Axio Imager.Z2.

#### 2.3.4. LF-NMR

For seven days, each set of cement mortar samples was cured under typical circumstances. After exposure to UV light and without UV light for 28 days, the samples were cut into 20 × 20 × 15 mm test blocks and dried to a constant mass. The blocks were then saturated using a vacuum saturation machine. LF-NMR (Low-Field Nuclear Magnetic Resonance) was employed to obtain the relaxation curves of pore water in the concrete. The relaxation time reflects the distribution of pore sizes within the concrete, with smaller pores resulting in shorter relaxation times [61]. The MesoMR12-060H-1 (manufactured by Niumai Co., Ltd., Suzhou, China) low-field nuclear magnetic resonance instrument was employed to measure the porosity as well as the pore size distribution of the test blocks.

#### 2.3.5. Mass Loss

The samples of cement mortar were cured under standard conditions for 7 days, after which their mass was measured daily at fixed times for a further 7 days, with measurements taken under two conditions: with and without UV radiation. The mass loss rate (WL) of the samples can be calculated using the following formula:

(3) $$W_{L}=\frac{M_{0}-M_{i}}{M_{0}}\times 100\%$$

where *M_0_* represents the initial mass of the sample (g) and *M_i_* denotes the measured mass of the sample on different days during the experimental period (g).

#### 2.3.6. Thermogravimetric Analysis (TG/DTG)

Thermogravimetric analysis was conducted on a Netzsch STA 449 F3 analyzer (Netzsch, Selb, Germany) to quantify the content of hydrated as well as carbonated phases. The samples were heated from 30 to 1000 °C at a rate of 10 °C/min with a nitrogen flow of 20 mL/min. Prior to measurement, the test surfaces of each specimen were cut and ground into powder. The powdered samples were then vacuum-dried at 60 °C for 24 h.

#### 2.3.7. Fourier Transform Infrared Spectroscopy (FTIR) Test

The FTIR spectrum was obtained with a Nicolet iS20 infrared spectrometer produced by Thermo Fisher Scientific (Waltham, MA, USA). The testing frequency range was set at 400–4000 cm^−^^1^ and the test mode was transmission. The samples were vacuum-dried at 60 °C for 24 h.

#### 2.3.8. XRD Analysis

The XRD patterns were recorded by an X-ray diffractometer using a Rigaku SmartLab SE (Tokyo, Japan) with a Cu Kα source (0.154 nm) in a 2θ range of 5–65°at a scan rate of 1 s/step. The samples were vacuum-dried at 60 °C for 24 h.

## 3. Results and Discussion

### 3.1. Influence of UV Radiation on the Appearance Quality of Fair-Faced Concrete

#### 3.1.1. Glossiness

**Figure 4:**
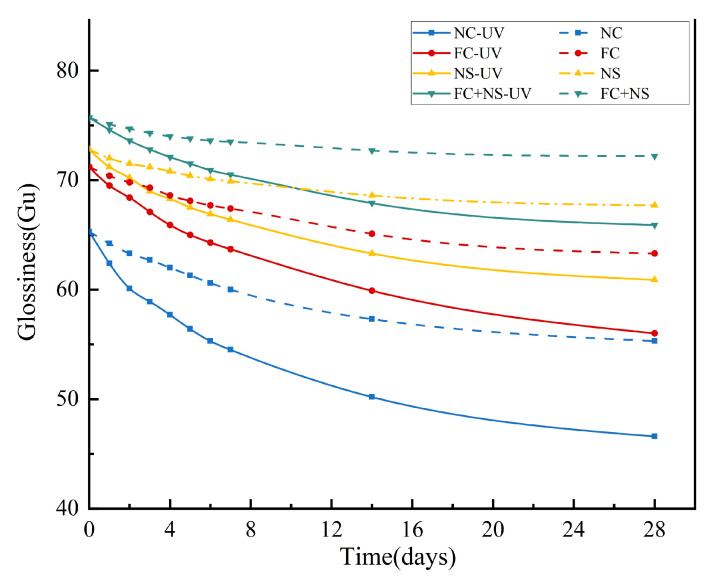
The glossiness of UV radiation and untreated samples.

Under the same humidity and temperature conditions, the reduction in the surface glossiness of each group of samples with and without UV radiation is illustrated in Figure 4. Table 3 indicates that the surface glossiness of the samples gradually decreased over time. The rate of glossiness decline generally exhibits an initial rapid decrease, followed by a slower decline. It is evident that the incorporation of NS and FC increases the surface glossiness of the samples. Among them, the fluidity of the mortar is enhanced by the addition of FC, resulting in a smoother surface for the molded mortar. Meanwhile, the ultra-fine particle size of NS enables it to effectively fill pores, thereby reducing overall porosity and increasing density. Furthermore, NS can react with hydration products to produce additional hydration products, thus accelerating the hydration process [62,63]. Collectively, these factors contribute to an increase in the glossiness of the mortar mixed with the admixture. However, when FC and NS are used together, the further improvement in surface glossiness may be attributed to the potential agglomeration effect of NS. Agglomerated particles may locally accumulate on the fair-faced concrete surface, leading to uneven coloration characterized by dark or light spots. These agglomerated regions could also act as stress concentration points, where uneven shrinkage during curing induces localized stresses, thereby increasing the probability of microcrack formation. Consequently, this phenomenon negatively impacts the surface glossiness of fair-faced concrete. In this scenario, the incorporation of FC serves as a lubricant, effectively mitigating nanoparticle agglomeration. This synergistic interaction between the two admixtures further optimizes surface properties and enhances glossiness.

It is evident that samples exposed to UV radiation exhibit a more significant reduction in glossiness. Additional data suggest that UV radiation increases both the porosity and maximum pore diameter of fair-faced concrete surfaces, as well as elevating the content of calcite. Collectively, these factors contribute to the diminished glossiness of fair-faced concrete. However, when compared to the NC group, the rate of glossiness reduction in samples containing admixtures was lower. Notably, the glossiness reduction rate for the FC+NS group decreased from 28.63% to 12.95% relative to the NC group, indicating that the combination of NS and FC not only enhances surface glossiness but also alleviates the detrimental effects of UV radiation on the glossiness of fair-faced concrete, thereby improving its durability.

#### 3.1.2. Surface Pore Distribution

Three parameters are utilized to evaluate the distribution of surface pores in fair-faced concrete: porosity, maximum pore diameter (max D), and the standard deviation of pore distribution (σ). The evaluation criteria are presented in Table 4 [64]. The standard deviation (σ) reflects the uniformity of pore distribution, with a higher σ indicating a less uniform pore distribution.

**Figure 5:**
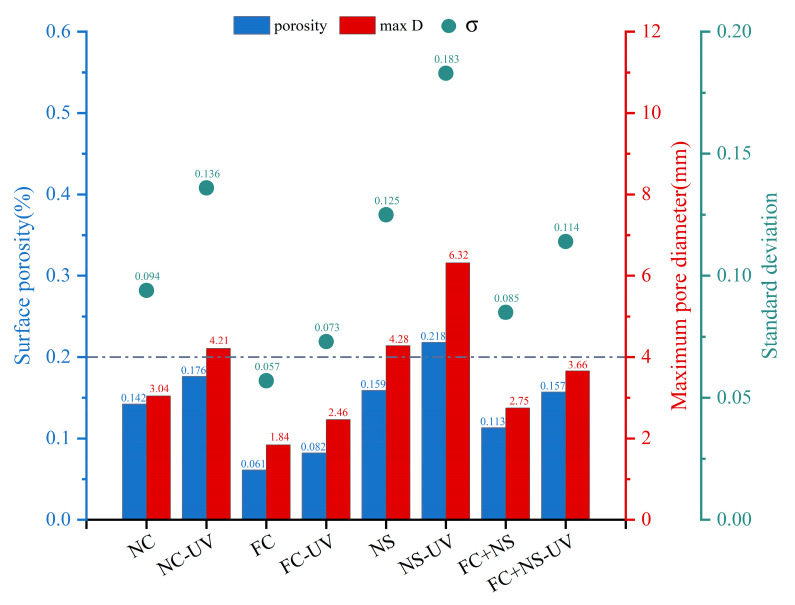
The surface pore distribution of UV radiation and untreated samples.

Figure 5 shows the surface pore distribution of each sample with and without UV radiation under the same humidity and temperature conditions. A demarcation line is drawn at a porosity of 0.2% as well as a maximum pore diameter of 4 mm. If the porosity and maximum pore diameter of the sample are below this line, the surface pore distribution of the fair-faced concrete is evaluated as excellent. The result clearly indicates that the surface pore area ratio, maximum pore diameter, and standard deviation of pore distribution for the samples subjected to UV radiation have all increased. This increase is attributed to the fact that UV radiation accelerates water evaporation [40], thereby enhancing internal porosity and resulting in a higher proportion of large pores and mesopores, which leads to an increase in surface pores. Notably, the increases observed in the FC and FC+NS groups are relatively minor, with both maintaining porosity below 0.2% and maximum diameters below 4 mm, even after 28 days of exposure to UV radiation. Conversely, the NC and NS groups were assessed as normal and unqualified, respectively, following UV radiation exposure.

Compared to the NC group, the FC group exhibited reduced surface porosity and smaller maximum pore size, attributable to the incorporation of water-based fluorocarbon paint. This additive enhanced flowability while decreasing plastic viscosity, thereby facilitating air escape during mixing and resulting in lower surface pore content.

In contrast, the NS group demonstrated inverse rheological behavior. The addition of nano-silica significantly impaired flow performance and increased plastic viscosity through two primary mechanisms: the extremely high specific surface area and pronounced particle aggregation tendency of nano-silica and the strong adsorption between NS surface active sites and flocculated C-S-H gel structures in hydration products. This adsorption effect particularly inhibited the deflocculation process of C-S-H structures, substantially elevating viscous resistance. During mixing and molding, the enhanced viscous resistance critically compromised bubble expulsion efficiency, ultimately leading to increased surface porosity and enlarged pore size.

#### 3.1.3. Surface Roughness

**Figure 6:**
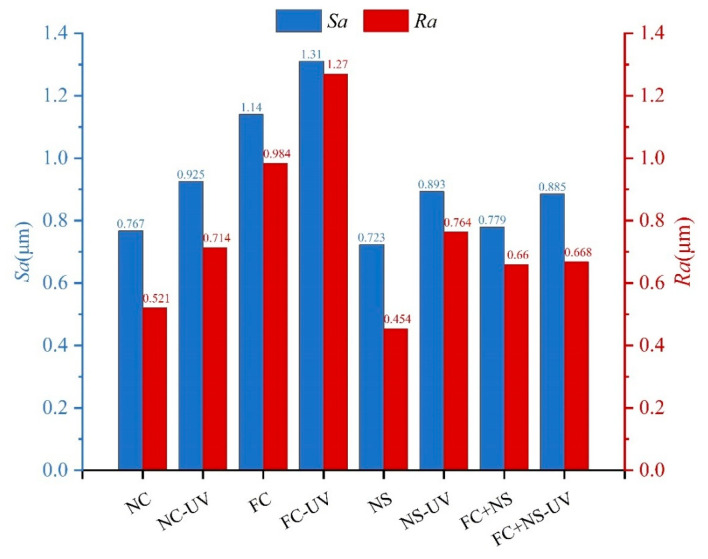
The surface roughness of UV radiation and untreated samples.

According to ISO 4287:1997 [59], surface roughness (Sa) and line roughness (Ra) are two critical parameters that serve as significant indicators for assessing the appearance quality of fair-faced concrete. Figure 6 illustrates the changes in surface roughness for samples exposed to 28 days of UV radiation compared to those without UV exposure under identical humidity and temperature conditions. Results indicate that both Sa and Ra increased after UV radiation. Among them, FC exhibited the highest values of Sa and Ra while NS and FS+NS demonstrated reductions in these parameters compared to NC. Although ultraviolet radiation can accelerate the early hydration process of cement-based materials, its photochemical reactions and thermal effects simultaneously promote the cleavage of hydroxyl bonds and the evaporation of free water. The reduction in free water content restricts the full progression of hydration reactions, ultimately leading to a decreased formation of hydration products. Consequently, specimens subjected to prolonged UV exposure exhibit a lower degree of hydration compared to those cured under natural conditions, accompanied by a decline in surface-layer densification. A denser surface layer corresponds to fewer surface pores and defects, as well as reduced surface roughness.

**Figure 7:**
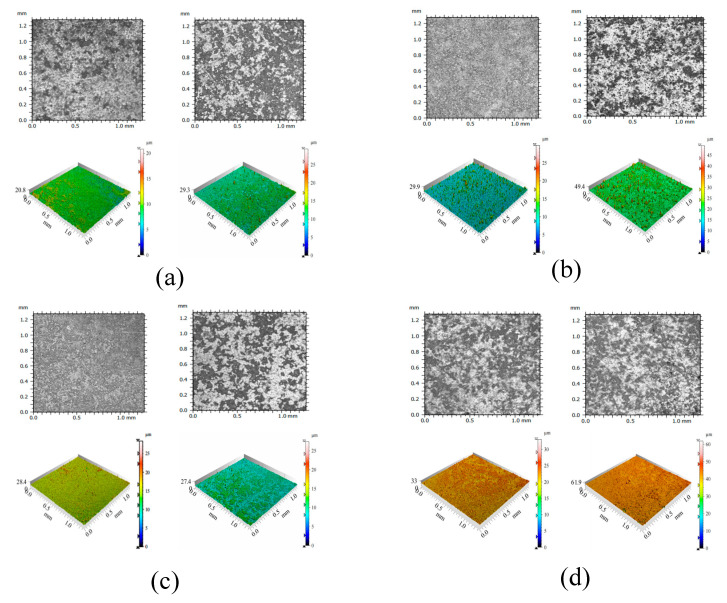
The microscopic morphology and optical profile images of UV radiation and untreated samples. (**a**) NC (**left**) and NC-UV (**right**); (**b**) FC (**left**) and FC-UV (**right**); (**c**) NS (**left**) and NS-UV (**right**); (**d**) FC+NS (**left**) and FC+NS-UV (**right**).

**Figure 8:**
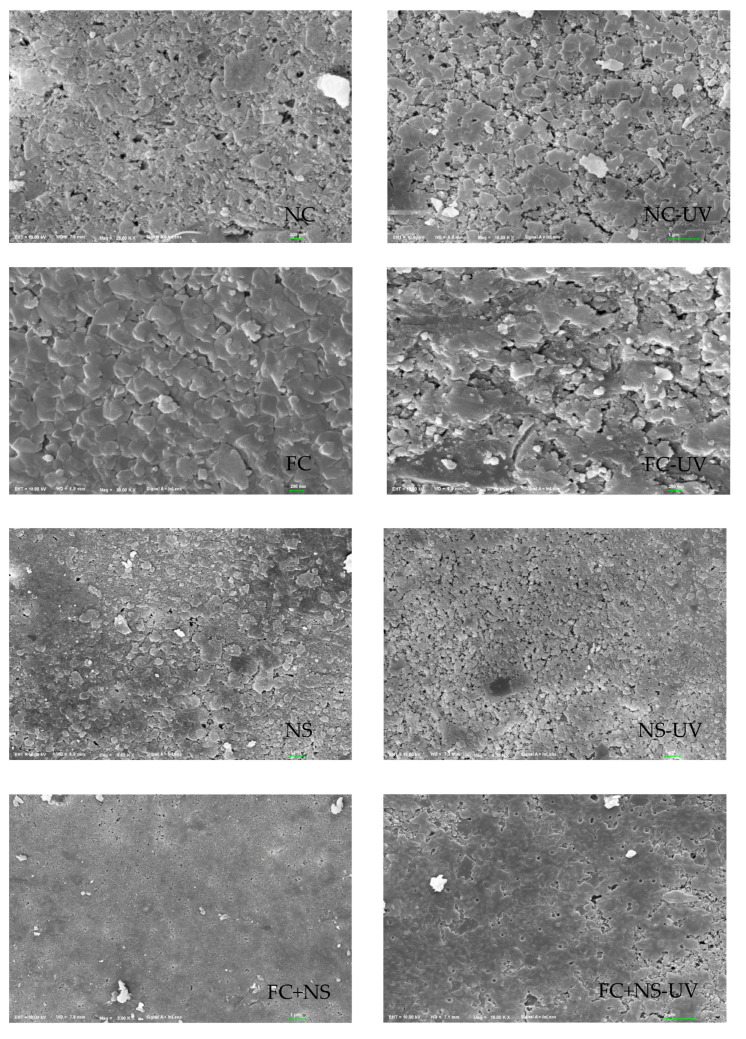
SEM images of 100 days exposed to UV radiation and untreated samples.

Figure 7 presents microtopography and optical profiles of test blocks under two environmental conditions. Each set of images on the right side corresponds to the UV radiation group. Figure 8 shows XRD images of test blocks under two environmental conditions. The micro-morphological analysis clearly reveals that the UV-irradiated samples exhibit a loosened surface structure with increased and unevenly distributed calcium carbonate formations, accompanied by localized agglomeration. This phenomenon primarily results from UV-induced thermal stress that initiates microcracking and surface delamination.

Due to the low thermal conductivity of concrete, a significant temperature gradient develops between the surface and interior, which cannot be rapidly equilibrated through thermal conduction. The mismatch in the coefficients of thermal expansion between the surface mortar and internal aggregates generates interfacial shear stresses, leading to microcrack formation. Crack propagation occurs when the released elastic strain energy equals or exceeds the surface energy required for new crack formation. When the cumulative energy released by the crack network surpasses the energy required for material detachment, surface spalling ensues.

The incorporation of FC increases surface roughness, whereas the addition of NS reduces it. NS not only fills surface pores but also reacts with Ca(OH)_2_ generated during cement hydration in a secondary reaction, producing additional C-S-H gel, thereby densifying the surface structure of fair-faced concrete. In contrast, the addition of FC enhances the fluidity of cement mortar, leading to an overall increase in porosity and mesopore quantity within the cementitious matrix, which exacerbates surface defects. As evident in Figure 8, after 100 days, the NS and FC+NS groups exhibit fewer surface cracks and a smoother texture compared to the NC group, whereas the FC group shows severe surface spalling and the highest degree of surface defects. These microscopic observations are consistent with the surface roughness measurements.

In summary, UV radiation can increase the surface roughness of fair-faced concrete. Meanwhile, UV radiation also leads to a decrease in surface glossiness, an increase in the surface porosity, and an enlargement of the maximum pore diameter, which significantly degrades the appearance quality of fair-faced concrete. Nevertheless, the FC+NS experimental group exhibited the highest surface glossiness under UV radiation, receiving an excellent evaluation for surface pore distribution and demonstrating lower surface roughness. This demonstrates that the combined use of these two admixtures not only improves the appearance performance of fair-faced concrete but also mitigates the aging effect of UV radiation on its surface.

### 3.2. Influence of UV Radiation on the Loss of Mass of Fair-Faced Concrete

**Figure 9:**
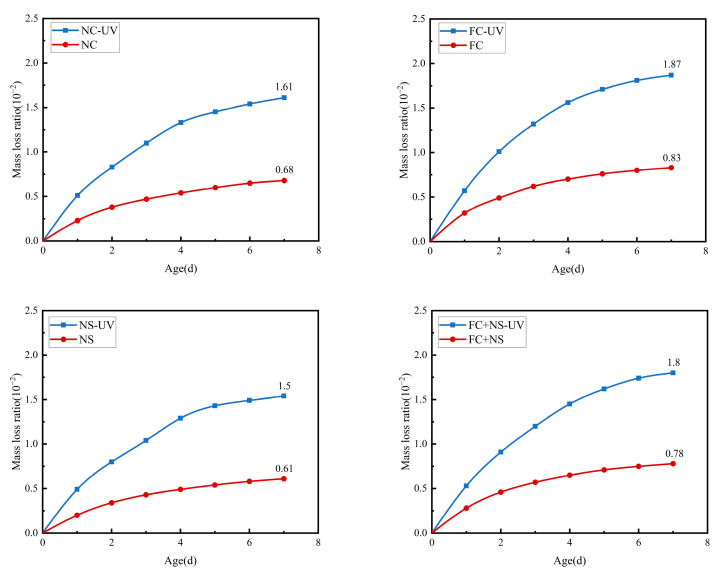
The mass loss of UV radiation and untreated samples.

Under the same humidity and temperature, the mass loss of each group of specimens with and without UV radiation during water evaporation is displayed in Figure 9 and Table 5. The results indicate that as the age of the samples increases, the mass loss rate gradually rises. Specifically, this rate exhibits an initial rapid loss followed by a deceleration. After each mortar reaches an age of 5 days, its mass loss rate tends to stabilize. Notably, the mass loss of each sample group subjected to UV radiation is significantly greater than that of the group not exposed to UV radiation. Consequently, UV light appears to enhance the mass loss rate of samples under consistent temperature and humidity conditions. This phenomenon is attributed to the ability of UV light energy to accelerate the motion of free water molecules [40], thereby increasing the rate of free water evaporation.

Further examination of mass loss rates across various sample components under two environmental conditions revealed that the NS group mortar exhibited the lowest mass loss rate, followed by the FC group with the highest rate, then the FC+NS group, and finally the NC group. One reason for this phenomenon is that NS possesses a very high specific surface area and surface energy, which can interact with the hydration products of cement-based materials, such as Ca(OH)_2_, to generate calcium silicate hydrate gel (C-S-H), thereby accelerating the hydration reaction. This interaction results in increased water demand, a reduced content of free water, and, consequently, a decreased amount of free water available for evaporation. Additionally, the inclusion of FC, a liquid fluorocarbon coating, enhances the flow properties of the mortar, thereby reducing its water demand and increasing the content of free water. Furthermore, when FC and NS are both combined in the mortar, FA effectively diminishes the tendency of NS to agglomerate due to its high surface energy, thereby alleviating the reduction in fluidity caused by NS.

### 3.3. Pore Structure Analysis

**Figure 10:**
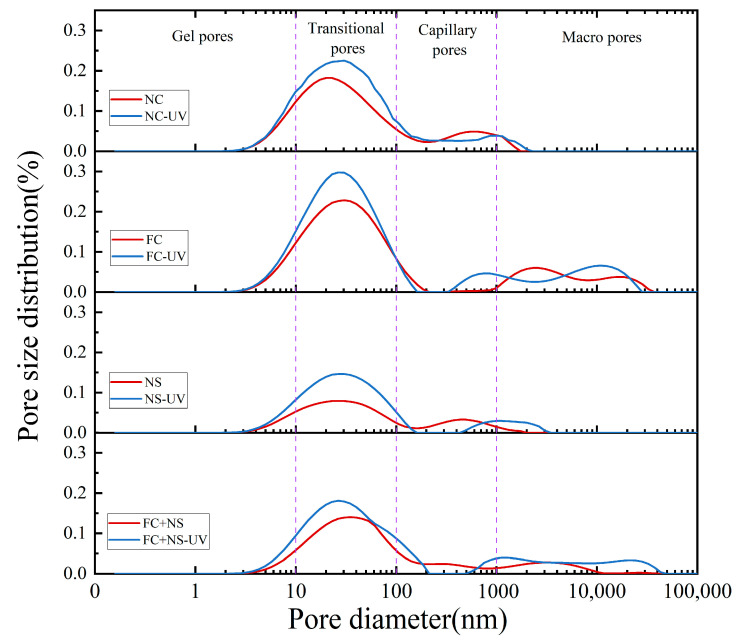
The pore size distribution of UV radiation and untreated samples.

**Figure 11:**
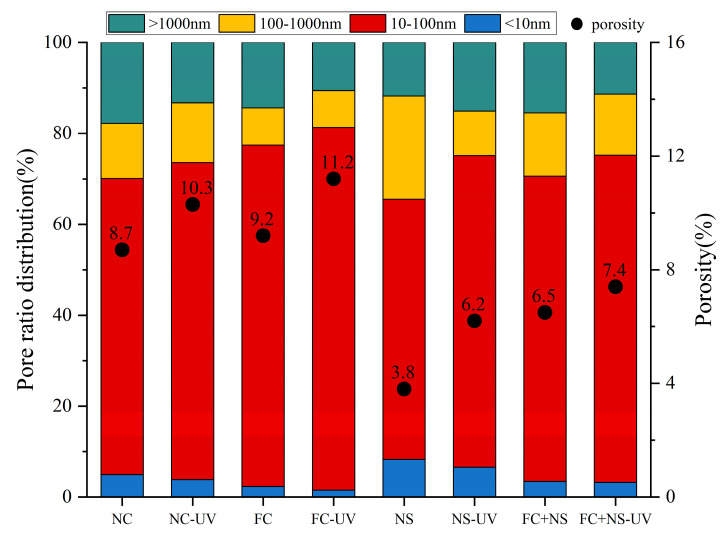
The pore ratio distribution and porosity of each group.

Concrete contains four types of pores, classified by size: gel pores (d < 10 nm), transitional pores (10 < d < 100 nm), capillary pores (100 < d < 1000 nm), and large pores (d > 1000 nm). LF-NMR can be utilized to determine the pore size distribution of each test block under two different environments, as shown in Figure 10. By integrating the curve of the pore size distribution, the proportion of pore sizes, as well as the porosity of the test block, can be obtained, as shown in Figure 11. The comprehensive results from both figures indicate that the porosity of each sample increases under UV radiation, with significant increases observed in the volume and proportion of transitional pores. This phenomenon is attributed to the radiation energy of UV accelerating the motion of free water molecules, ultimately increasing the rate of free water evaporation. The reduction in free water content leads to decreased moisture within the pores of the concrete, which, in turn, increases pore size. Additionally, the decrease in free water slows the hydration reaction process, resulting in a reduced quantity of hydration products within the pores, thereby making them more difficult to fill.

By comparing the pore size distributions among groups with different admixtures, it can be observed that both the NS group and the FC+NS group exhibit reduced porosity and the most probable radius compared to the NC group while the porosity of the FC group increases. This finding aligns with the previously discussed appearance quality performance of the samples. A decrease in porosity indicates an enhancement in overall compactness, resulting in a smoother and more polished appearance of the fair-faced concrete surface. Furthermore, the reduction in porosity leads to fewer surface pores, lower surface roughness, and increased surface glossiness.

### 3.4. Chemical Bond Analysis

**Figure 12:**
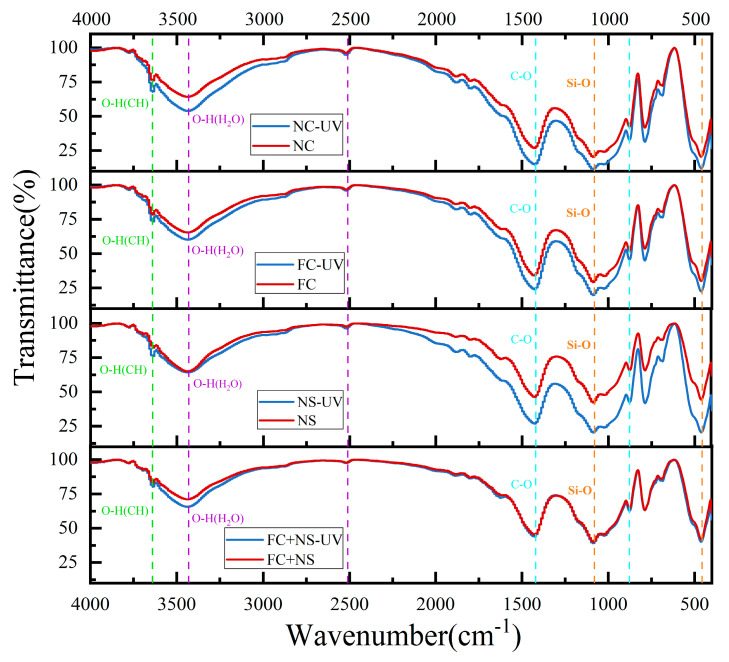
The FTIR spectra of each group.

The FTIR spectra of specimens with varying admixtures in two conditions are presented in Figure 12. The following are the primary chemical bonds identified in cement-based materials, as outlined by references [65,66]: (1) The peak at 3640 cm^−1^ corresponds to the O-H stretching vibration of calcium hydroxide (CH). (2) The broad band in the range of 3600–3100 cm^−1^ and the peak at 2510 cm^−1^ are linked to the O-H stretching vibration of water in the cement matrix. (3) The carbonate (CO_3_^2−^) peaks at 1420 cm^−1^ (C-O symmetric stretching vibration) and 877 cm^−1^ (out-of-plane bending vibration) are associated with calcite (a polymorph of CaCO_3_). In contrast, the FTIR peaks for the vaterite and aragonite phases are not clearly discernible. This is because the FTIR bands of vaterite (at 856 cm^−1^) and aragonite (at 876 cm^−1^) overlap with those of calcite and their intensities are significantly weaker. (4) The Si-O stretching vibration in calcium silicate hydrates (C-S-H) is responsible for the broad band in the 1200–800 cm^−1^ range and the peak at 456 cm^−1^.

Infrared spectroscopy patterns reveal that the primary chemical bond types in each group are similar under both environmental conditions, indicating that UV radiation does not induce new chemical bond formation or lead to the disappearance of existing ones in cement-based materials. To further investigate whether UV radiation affects the gel phase types of cement-based materials, X-ray diffraction (XRD) will be utilized. When comparing mortar samples subjected to the two environments, UV-irradiated samples exhibit significantly higher CO_3_^2−^ peak intensity (1420 cm^−1^ and 877 cm^−1^) than non-irradiated samples, except for the FC+NS group. The NC group exhibits the highest CO_3_^2−^ peak intensity, while the NS and FC groups show diminished peak intensities. The FC+NS group registers the lowest peak intensity. The incorporation of FC and NS mitigates the acceleration of carbonation reactions induced by UV radiation, thereby enhancing resistance to UV aging, as evidenced by trends in glossiness and surface roughness.

Figure 12 also indicates that the Si-O wavenumber broadband (1200–800 cm^−1^) does not display significant shifts under UV radiation, suggesting a stable degree of polymerization of the C-S-H gel. However, the UV-irradiated samples exhibit more pronounced signals within this range, which may be linked to accelerated carbonation reactions and a reduction in free water.

### 3.5. Hydration Process and Hydrate Phase Analysis

#### 3.5.1. XRD

**Figure 13:**
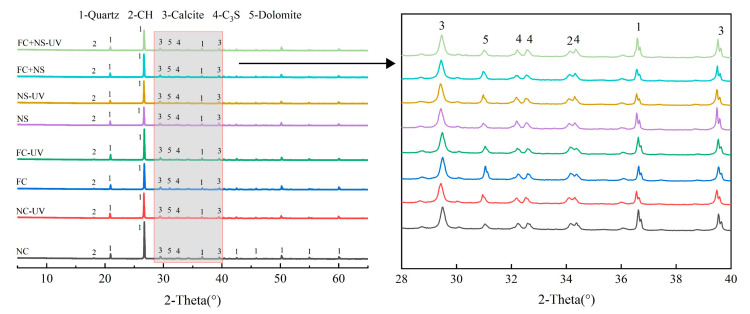
The XRD spectra of each group.

To examine the impact of UV radiation on the composition and types of hydration products in fair-faced concrete, XRD spectra from each sample group, stored in two different environments for 28 days, were analyzed. The phase types are displayed in Figure 13. Fair-faced concrete requires a significant amount of fine quartz powder to enhance its surface quality, resulting in prominent quartz peaks in the XRD spectra. The peaks beyond 40° are attributed solely to quartz and are only marked in the curve for NC. Among the identified phases, Ca(OH)_2_, calcite, and dolomite are hydration products generated through cement hydration and carbonation while C_3_S is the primary constituent of unreacted cement particles. The formation of dolomite is attributed to the high magnesium ion content in white cement.

The results presented in Figure 13 indicate that the peak positions for the hydration products of cement mortar in both environments are identical and the peak positions for mortars with different admixtures are also consistent. This suggests that UV radiation and the two types of admixtures do not induce new chemical reactions in the silicate system to produce new hydration products. Due to the high peak intensity of quartz, the peaks of calcite are not prominent in the XRD spectra. However, a comparison of peak intensities reveals that samples exposed to UV radiation exhibit higher peak intensities than those not exposed, which is consistent with the results from FTIR and TGA.

#### 3.5.2. TG/DTG

**Figure 14:**
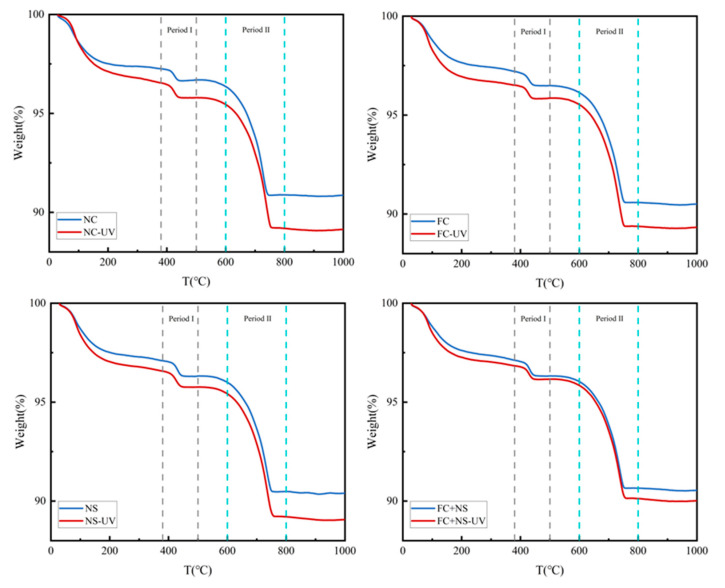
The TG curves of each group.

**Figure 15:**
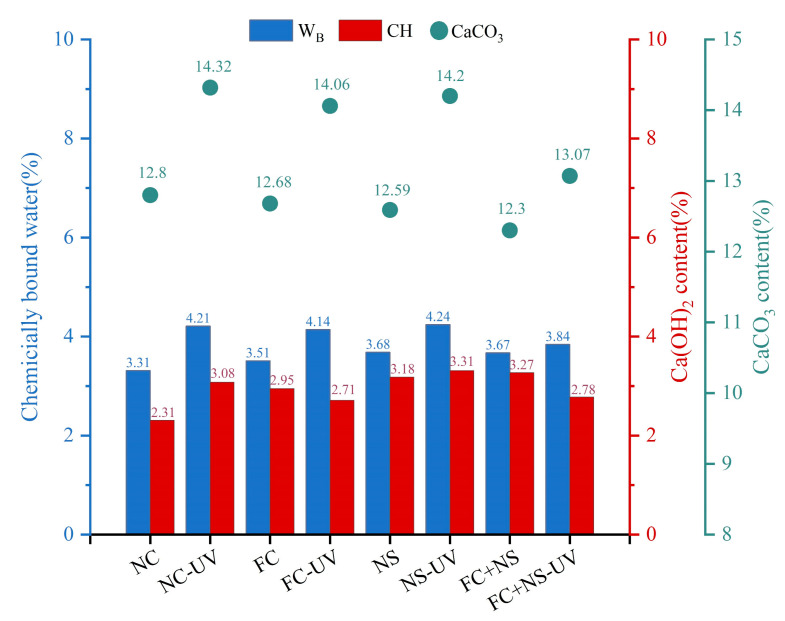
The chemically bound water, Ca(OH)_2_, and CaCO_3_ content of each group.

TGA was utilized to quantitatively assess the levels of hydration and carbonation on the surface of the specimens. Figure 14 presents the TGA plots of cement specimens containing various admixtures subjected to two different environments. Significant weight losses were observed during Period I and Period II, corresponding to the breakdown of the hydrated product calcium hydroxide (CH) as well as the decomposition of the carbonation by-product calcium carbonate (CaCO_3_), respectively. The weight loss observed prior to Period I is primarily associated with the breakdown of hydration products, including calcium-silicate-hydrate (C-S-H), ettringite (AFt), monosulfoaluminate (AFm), and portlandite. Additionally, Figure 14 also indicates that the content of calcium carbonate on the sample surface increased significantly following UV radiation, along with an increase in hydration products. In Figure 15, we quantitatively analyzed the chemically bound water, CH, as well as the calcium carbonate content of various specimens under two environmental conditions.

As shown in Figure 15, the content of calcium carbonate in the UV-radiated test samples is higher than that in the untreated control group, indicating that UV radiation significantly increases the carbonation degree of the specimen surface. Meanwhile, it can be observed that the content of calcium carbonate decreases in both environments when either FC, NS, or both are incorporated. Among them, NS exhibits a smaller decrease, followed by FC, while the FC+NS group shows the largest reduction, with the calcium carbonate content dropping from 14.32% to 13.07% compared to the NC group. This suggests that both admixtures have the effect of inhibiting the carbonation reaction of fair-faced concrete under UV radiation, thereby protecting the surface of fair-faced concrete from UV-induced aging as well as maintaining the durability of the surface quality. Furthermore, the simultaneous addition of both admixtures demonstrates a synergistic effect, significantly enhancing the inhibition of carbonation, which aligns with the findings from previous appearance quality testing.

The degree and pace of hydration can be directly reflected in the amount of calcium hydroxide, as well as chemically bound water, present. Observations indicate that the quantity of chemically bound water in the samples after UV radiation has increased, indicating that the quantity of hydration products has increased under UV radiation. This phenomenon can be attributed to the reaction of calcium hydroxide in the hydration products, which forms calcium carbonate following carbonation. UV radiation is believed to accelerate this carbonation reaction, resulting in the continuous deposition of calcium carbonate on the surface. According to principles of chemical reactions, this also accelerates the hydration reaction and increases the amount of hydration products, suggesting that UV radiation during the initial hydration stage can enhance the hydration rate. However, it is important to note that UV radiation also accelerates the evaporation of free water. According to the study by Wang et al. [50], after the hydration process is fully completed, the overall content of hydration products in the irradiated group is slightly lower compared to the non-irradiated group. A comparison between Groups NS and NC reveals that both the chemically bound water content and the CH levels in Group NS have increased. This increase can be attributed to the high specific surface area and surface energy of NS, which serve as nucleation centers for crystal growth and enhance the deposition of hydration products on the surfaces of cement particles. Furthermore, NS can react with the hydration products of cement-based materials, specifically Ca(OH)_2_, to form calcium silicate hydrate gel (C-S-H), thereby accelerating the hydration reaction [63]. The elevated levels of Ca(OH)_2_ in the hydration products due to the presence of NS also lead to an increased quantity of calcium carbonate, resulting in the impact of NS on inhibiting UV aging not being particularly significant. In Group FC, the rise in chemically bound water content may be attributed to the addition of FC, which increases the free water content in the system, resulting in the production of more hydration products during the hydration reaction.

### 3.6. Principle of UV Radiation Accelerating the Carbonation Reaction

As cement clinker hydrates in cement-based materials, Ca(OH)_2_ and calcium silicate hydrate (C-S-H) are generated, both of which are vulnerable to carbonation. Additionally, unhydrated clinker components, such as C_3_S and C_2_S, are also capable of undergoing carbonation. These compounds are located on the solid surface of cement-based materials. In a humid environment, a water film forms on this solid surface, allowing carbonatable substances, such as Ca(OH)_2_, to gradually dissolve into the surface water film. Carbon dioxide (CO_2_) from the atmosphere slowly infiltrates the surface and pores of the cement-based material, subsequently dissolving into the liquid phase. In the liquid film, Ca(OH)_2_ reacts with CO_2_ to form calcium carbonate, which precipitates on the surface and within the pores of the cement-based material. This process constitutes the carbonation of cement-based materials [67,68].

These compounds are situated on the solid surface of cement-based materials. In a humid environment, a water film develops on this solid surface, enabling carbonatable substances, such as Ca(OH)_2_, to gradually dissolve into the surface water film. CO_2_ from the atmosphere slowly infiltrates the surface and pores of the cement-based material, subsequently dissolving into the liquid phase. Within this liquid film, Ca(OH)_2_ reacts with CO_2_ to form calcium carbonate, which precipitates on the surface and within the pores of the cement-based material. This process constitutes the carbonation of cement-based materials.

**Figure 16:**
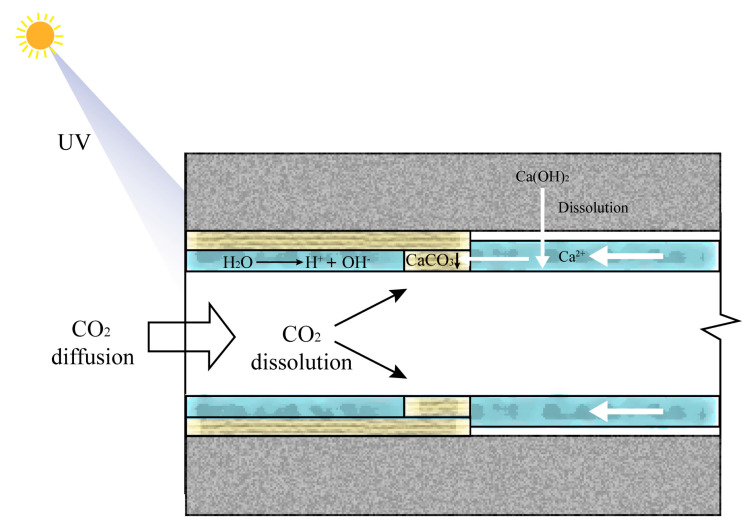
The principle of UV radiation accelerating the carbonation reaction.

The carbonation process of cement-based materials involves diffusion, dissolution, and precipitation reactions. These stages can be categorized into the diffusion and dissolution of CO_2_ and Ca(OH)_2_ from the gaseous phase into the liquid state, followed by the precipitation of the reaction product, calcium carbonate. UV light serves as a catalyst, accelerating the diffusion of CO_2_ and Ca(OH)_2_ from the gas phase to the liquid phase, thereby accelerating the carbonation reaction, as illustrated in Figure 16. The process can be described as follows:

H_2_O + UV → OH^−^ + H^+^

CO_2_ + 2OH^−^ → CO_3_^2−^ + 2H_2_O

Ca(OH)_2_ + 2H^+^ → Ca^2+^ + 2H_2_O

Ca^2+^ + CO_3_^2−^ → CaCO_3_↓

Simultaneously, UV radiation energy can enhance the motion of free water molecules, resulting in an increased evaporation rate of free water. Based on data on pore size distribution, UV radiation can increase the overall porosity of concrete, particularly by increasing the area of large pores. This enlarged pore area facilitates the entry of CO_2_ into the interior of the concrete, thereby accelerating the carbonation reaction [69].

The mechanism by which the incorporation of FC inhibits UV-radiation-induced aging is attributed to the high-bond energy (485 kJ/mol) and the remarkable stability of the C-F bond. FC can transmit more than 95% of medium and long wavelengths in the UV spectrum (220–400 nm) of sunlight. Only UV light with wavelengths of 220 nm or shorter can break the C-F bond. However, the proportion of these short-wavelength UV rays in sunlight is minimal and easily absorbed by the ozone layer. In contrast, NS demonstrates excellent optical properties, capable of reflecting and scattering UV light within the UV wavelength range, thereby reducing the amount of UV light that reaches the material. Due to its nanoscale dimensions, NS effectively enhances the reflectivity of the material surface, obstructing direct UV radiation to the substrate. Furthermore, the micro-filling effect, pozzolanic effect, and accelerated nucleation effect of NS can contribute to increasing the density of cement-based materials, reducing porosity, decreasing the proportion of large pores, and impeding the ingress of CO_2_ into the concrete interior, therefore diminishing the carbonation reaction.

In summary, UV radiation generally impacts the appearance quality of fair-faced concrete by accelerating the carbonation reaction and increasing porosity. These two admixtures serve to slow the carbonation reaction and fill internal pores, thereby mitigating the damage to appearance quality caused by UV radiation.

## 4. Conclusions

This study examines the appearance quality, mass loss, pore size distribution, and microstructure of mortar containing various admixtures under two conditions: with and without UV radiation. The experimental results lead to the following conclusions:

(1) Under UV radiation, the appearance quality of fair-faced concrete deteriorates, evidenced by decreased surface glossiness, increased surface porosity and maximum pore diameter, and a reduction in surface roughness;

(2) UV radiation accelerates water evaporation, resulting in increased porosity. Additionally, it can expedite carbonation and early hydration reactions, leading to the formation of more calcite on the surface of the sample. These factors contribute to a significant reduction in the appearance quality of fair-faced concrete;

(3) The modified fair-faced concrete with admixtures demonstrated significant performance improvements compared to the control group: under identical ultraviolet radiation conditions, its carbonation degree was markedly reduced, effectively mitigating the deterioration of surface quality caused by carbonation reactions. However, single-admixture modifications exhibited inherent limitations—nano-silica increased surface porosity and maximum pore size while water-based fluorocarbon paint elevated surface roughness;

(4) When these two admixtures were combined, the material exhibited outstanding comprehensive performance: surface roughness decreased by 6.4%; surface porosity remained below 0.2%, with a maximum pore size under 4 mm; and the surface gloss attenuation rate dropped from 28.63% to 12.95%. The synergistic effect of the dual admixtures enabled the composite-modified concrete to achieve excellent initial surface quality, superior long-term appearance retention, and enhanced UV resistance.

## Figures and Tables

**Table 1:** The chemical composition of cement, MK, and LP (wt.%).

Raw Materials	Chemical Composition								
	SiO_2_	CaO	Al_2_O_3_	Fe_2_O_3_	SO_3_	MgO	Na_2_O	K_2_O	Others
PW	22.76	62.45	5.28	0.72	2.68	1.48	0.37	0.46	3.80
MK	48.82	0.83	42.27	0.00	0.15	0.26	0.07	0.05	7.56
LP	7.97	86.98	2.31	1.17	0.05	0.15	0.00	1.01	0.66

**Table 2:** Constituents of the concrete mixtures.

Sample	Cement (kg/m^2^)	MK (kg/m^2^)	LP (kg/m^2^)	Quartz Sand (kg/m^2^)	Quartz Flour A (kg/m^2^)	Quartz Flour B (kg/m^2^)	Quartz Flour C (kg/m^2^)	Quartz Flour D (kg/m^2^)	Water (kg/m^2^)	Water Reducing Agent (%)	Defoaming Agent (%)	NS (kg/m^2^)	FC (kg/m^2^)
NC	544	96	128	256	256	256	128	128	174.7	1	0.05		
FC	544	96	128	256	256	256	128	128	174.7	1	0.05		8.96
NS	544	96	128	256	256	256	128	128	174.7	1	0.05	4.98	
FC+NS	544	96	128	256	256	256	128	128	174.7	1	0.05	4.98	8.96

**Table 3:** The glossiness of each group after 28 days of exposure.

Group	NC	NC-UV	FC	FC-UV	NS	NS-UV	FC+NS	FC+NS-UV
Glossiness	55.8	45.1	65.2	56.1	68.9	63.7	74.4	68.1

**Table 4:** Standard for evaluating surface pore grades of fair-faced concrete [63].

Level	Excellent	Normal	Unqualified
standard	porosity < 0.2% and max D < 4 mm	porosity < 0.2% and 4 mm < max D < 8 mm	porosity > 0.2% or max D < 4 mm

**Table 5:** The mass loss of each sample after 7 days of exposure.

Group	NC	NC-UV	FC	FC-UV	NS	NS-UV	FC+NS	FC+NS-UV
Mass loss ratio (%)	0.68	1.61	0.83	1.87	0.61	1.5	0.78	1.8

## Data Availability

The original contributions presented in this study are included in the article. Further inquiries can be directed to the corresponding author.

## Abbreviations

The following abbreviations are used in this manuscript:

UV	Ultraviolet
FC	Fluorocarbon paint
NS	Nano-silica
FFC	Fair-faced concrete
